# Efficacy of Sublingual Immunotherapy versus Subcutaneous Injection Immunotherapy in Allergic Patients

**DOI:** 10.1155/2012/492405

**Published:** 2012-02-20

**Authors:** Diego Saporta

**Affiliations:** Associates in ENT & Allergy, PA 470 North Aveue, Elizabeth, NJ 07208, USA

## Abstract

While it is generally accepted that Subcutaneous Injection Immunotherapy (SCIT) and Sublingual Immunotherapy (SLIT) are both efficacious, there is not yet a significant amount of information regarding their comparative efficacy. In this paper, we performed a retrospective chart review and compared treatment results in two groups of patients (both with nasal allergies with or without asthma) that were treated either with SCIT or SLIT. Both treatment modalities were found to be of similar efficacy.

## 1. Introduction

Allergic disease is an increasingly prevalent problem affecting up to one-third of the general population in industrialized countries. Immunotherapy is a treatment modality that can modify the immunological response of the allergy sufferer so that the affected individual will stop reacting to involved allergens. Immunotherapy is indicated for the treatment of allergic rhinitis (AR) and asthma [1], and it may prevent development of asthma in patients with AR [1, 2].

Immunotherapy can be administered by different routes amongst which we find injectable and oral vaccines. Injectable vaccines refers to the classical subcutaneous injection immunotherapy (SCIT) usually known as “allergy shots.” Oral vaccines refer to sublingual immunotherapy (SLIT) where the allergens are administered as drops to the sublingual area even though the term oral vaccines may also include allergy tablets [3].

The purpose of this study is to compare the efficacy of treatment results in patients with nasal allergies, with or without asthma, that were treated with either one or the other of these two treatment modalities: SCIT or SLIT.

There is a voluminous body of scientific evidence that proves that these two treatment modalities are efficacious for the management of allergic conditions but the issue of these two modalities having similar efficacy has not yet been fully addressed. A review of the literature reveals only a few articles that directly address this issue [4–10]. In five of these reports [5–9] SCIT and SLIT are found to be equally effective. In one report [4] SCIT is found to have better results, and one report [10] finds both equally effective for AR patients but SCIT more effective for asthmatic patients. In our own experience, SLIT and SCIT appear to be of similar efficacy [11] In this report the efficacy of one will be compared against the other.

SCIT is a well-established treatment modality that has been successfully used for many decades and is relatively well tolerated. Occasionally patients can develop severe reactions that very rarely can result in mortality [12].

SLIT is also a very old treatment modality (earliest description is from 1900) and yet, while commonly used in Europe, it is still not well established in the USA [13]. Over the last 20 years the European medical community produced a large amount of high-quality evidence suggesting that SLIT is safer than SCIT [14, 15]. While no single case of mortality has ever been reported with SLIT [12, 16] this is not the case with SCIT [17, 18]. SLIT is so safe and easy to administer that patients treat themselves at home [19].

## 2. Methods

This study constitutes a retrospective, consecutive chart review of allergy patients treated by the author at his private office. The charts of active patients were alphabetically reviewed to determine eligibility. Inclusion criteria were as follows: a patient of any age with nasal allergies with or without asthma that was treated with immunotherapy for at least for 6 months and had at least 2 complete evaluations. A complete evaluation implies symptom scoring, evaluation of medication use, and determination of the peak flow meter (PFM) value. These evaluations are done every 3–6 months as treatment progresses. Because evaluations depend on patient's cooperation not all the patients had the same number of evaluations, but any patient that was considered a candidate had to have 2 evaluations as a minimum. We compared the first evaluation (pretreatment) and the last evaluation the patient had just at the time of inclusion for the study. These were considered pretreatment and posttreatment evaluations. The symptoms in the pretreatment evaluation and the amount of medications the patient was taking at that time reflect how the patient was doing without immunotherapy treatment.

*Ethical Considerations*. Subjects' privacy was respected by collecting and recording data in such a way that the subjects could not be identified, directly or indirectly, through identifiers linked to the subject. In other words, a patient's confidentiality would be protected by entering data in a simple spreadsheet with nonspecific identifiers as patient no. 1, patient no. 2, and so forth with subsequent refiling of the patient's chart, according to usual procedure. The content of the spread sheet became anonymous and ready for statistical analysis.

### 2.1. Decision to Use SCIT or SLIT

After discussing with patient about their allergies and advising about environmental modification maneuvers a discussion about treatment options including immunotherapy follows. In our office SCIT or SLIT is used to treat patients with inhalant allergies with or without bronchial involvement. The decision to use one or the other is sometimes made by the patient, sometimes advised by the treating physician. Economical considerations, living far from the office, busy schedule, or “needle phobia,” are examples of when a patient may chose SLIT. Having severe asthma, being a very young patient or having medical problems that may render administration of SCIT risky are examples of why the treating physician will advise SLIT.

### 2.2. Testing and Treatment Administration

All patients were tested using a fivefold intradermal dilution skin test (IDT) as taught by the AAOA [20, 21]. The test includes several panels: dust, dander, epidermals, molds, and pollens for our geographic area (Table 1).

Standardized antigens were used for testing and treatment whenever these were available; otherwise weight/volume antigen extracts were used [22].

After identifying the minimally reactive antigen concentration (meaning first reactive wheal) for each of the patient's reactive allergens, SCIT vials or SLIT bottles were formulated including all of the positive results (reactive allergens in the intradermal test) in the treatment mixture. Patients on SCIT were treated according to AAOA guidelines [21, 23]. Patients on SLIT were treated according to a previously published protocol [11] where the dose is slowly advanced from 1 drop per day to 5 drops per day until attaining the most concentrated mixture in the SLIT bottle. The formulation was the same for both injectable and oral vaccines.

### 2.3. Amount of Antigen Delivered

While the concentration of antigens is exactly the same for both SCIT and SLIT but SLIT is administered daily [11], patients on SLIT will receive a larger amount of antigen each week than those treated with SCIT. The injectable vials are mixed with a volume of 5.0 mL. The SLIT bottles are mixed with 7.5 mL. If we consider a single allergen, for example, *Dermatophagoides pteronyssinus* (DP), standardized dust mite DP has a concentration of 10,000 AU/mL containing 68 mcg/mL of Der p 1 and 71 mcg/mL of Der p 2 antigens [22]. If the minimally reactive antigen concentration occurred at dilution no. 3 and dose was advanced until mixing a vial from manufacturer's concentrate, the cumulative dose this patient would receive weekly by SCIT would be 200 AU per week, while a patient treated by SLIT would receive 464 AU per week [11]. As stated before, the initial allergen concentration in both SCIT and SLIT is the same: 80 AU/mL as in both circumstances the extract (with 10.000 AU/mL) will be diluted 125 times. After one year of treatment the patient on SCIT would receive 9680 AU and the patient treated by SLIT would receive 21149 AU or 2.18 times more allergen [11].

### 2.4. Sample Comparison in reference to Allergen Reactivity

A chi-square test was applied for the following allergens: dust mite, cat, roach, mold, tree-pollens, grass-pollens, and weed-pollens for both groups, SCIT and SLIT.

### 2.5. Asthma Diagnosis

Asthma diagnosis was based on the presence of recurrent cough, chest tightness, SOB, or wheezing [24], having a spirometry consistent with airflow obstruction or having the symptoms respond to the administration of a short-acting broncho-agonist (SABA).

### 2.6. Scoring

Recorded symptoms included runny nose, sneezing, nasal obstruction, itchy eyes, itchy ears, cough, shortness, and wheezing. These were scored according to Fell's method [25] with a numerical analog from 0 through 3 as follows:

0 = symptom not present,
1 = symptom is mild,
2 = symptom is moderate,
3 = symptom is severe.

Medication use was also evaluated on a similar numerical scale as follows:

0 = medication is not being used,
1 = medication is being used once a week or less,
2 = medication is being used 2–3 times per week,
3 = medication is being used 4 or more times per week.

Medications were generically grouped as allergy pills, intranasal steroids (INSs), and short-acting broncho-agonists (SABAs) in the case of asthmatic patients.

The value of the PFM determination was used as the parameter to be recorded at each patient's encounter.

## 3. Results

Ninety-three charts met the inclusion criteria, 50 on SCIT and 43 on SLIT. Among the 50 patient's on SCIT, 20 (40%) were male, 30 (60%) female ranging in age from 2.33 to 75 years (mean 45 ± 17.8 SD). This compared to 43 patients on SLIT of whom 21 (49%) were male, 22 (51%) female ranging in age from 1.66 to 75 years (mean 35 ± 20.8 SD). There are no statistical differences between the demographics of both groups. Analysis of covariance for the dependent variables for which a significant pre/posttreatment by treatment modality interaction effects was obtained did not reveal gender or age to account for significant dependent variable variance; in other words the results were not affected by age or gender so both groups can be considered homogeneous. Both groups were also compared in reference to test results. A chi-square test was applied for the following allergens: dust mite, cat, roach, mold, tree-pollens, grass-pollens, and weed-pollens. Results indicate that there are no statistical differences between both groups (at the *P* < 0.05 level); therefore in their reactivity to allergens both groups can also be considered homogeneous.

There were 3 children <12 years on SCIT (mean 7.8 years) versus 11 on SLIT (mean 6.9 years). Ten (20%) SCIT patients had asthma versus 12 (28%) on SLIT. Thus a greater percentage of asthmatics (12/22 or 55%) and more children under 12 years of age (11/14 or 79%) were on SLIT. Length of treatment for the SCIT group was 12 to 86 (mean 31 ± 18.7 SD) months and for the SLIT group was 10 to 32 (mean 19 ± 6.3 SD) months.

For all patients the pre- and posttreatment averages for each symptom, medication use, and PF value were statistically compared through the use of repeated measure analysis of variance (ANOVA). The results for the two treatment modalities (SCIT versus SLIT) were also compared using the between-subjects factor of the ANOVA (Table 2). The same analyses were completed for medication use (Table 3). For the PF evaluation the pre- and post-treatment values were compared (Table 4).

### 3.1. Symptom Results

In Table 2 the mean value for each symptom score before treatment and at the time of data collection is shown for both treatment modalities. The result of the test of significance is shown for each symptom within each treatment modality (paired *t*-test). Lastly, the result of the statistical analysis comparing symptom improvement with one or the other treatment modality is shown.

All symptoms had significant improvement with both treatment modalities. Shortness of breath and wheezing had significant improvements at *P* < 0.05 for both treatment modalities. The remaining symptoms had a significant improvement at *P* < 0.001 for both treatment modalities.

Wheezing and coughing were the only symptom scores which seemed to respond better to either SCIT (coughing slightly better, *P* = 0.037) or SLIT (wheezing slightly better, *P* = 0.024), though both symptoms significantly improved regardless of treatment modality. For the remaining symptoms there was no significant difference between both treatment modalities.

### 3.2. Results of Medication Use

Both SCIT and SLIT provided equally significant reduction in use of medication (*P* < 0.001) including allergy pills, INS, and, to a slightly lesser but still significant degree, SABA (Table 3) but without no significant difference between both treatment modalities.

### 3.3. Results of Changes in PFM Values

PF value before treatment and at the time of the last patient evaluation is shown in Table 4. Both treatment modalities were equally effective in achieving a significant increase in PF values (*P* < 0.001) but there was no significant difference between both treatment modalities.

## 4. Discussion

This paper is a retrospective chart review and as such lacks the rigor of a prospective randomized study with a placebo control group which is very difficult to do in a private office setting. While an analysis of covariance is useful, it is not a perfect solution. A future, larger-scale study should be planned to include the above design characteristics.

We observed that patients usually come to the office already using one or more allergy medications. This study, like others, demonstrates that immunotherapy, whether SCIT or SLIT, will lead to the reduction of medication use for AR and/or asthma. It was not the purpose of this paper to evaluate the effect of medications on allergy symptoms but rather to compare the effects of SCIT versus SLIT on medication use. Both treatment modalities resulted in the reduction of antihistamines, inhaled nasal steroids, and SABAs.

The slight imbalances in demographic characteristics between the groups on SCIT versus SLIT were not statistically significant and did not affect the statistical results. The reason why there are more young patients and more asthmatic patients in the SLIT group can be explained by the fact that SLIT is safer and easier to administer therefore it is suggested more frequently for these difficult-to-manage patients. Indeed we would have expected a much more pronounced difference; yet fewer than expected chose SLIT because it is not covered by insurance.

Patients on SCIT have been treated for a longer period of time because SLIT was added to our practice later than SCIT.

The improvement of the asthmatic symptoms wheezing and SOB and the decrease in SABA use were significant at *P* < 0.05 yet because of sample size this is not as strong as the improvement in other symptoms or medications that had an improvement at the level of *P* < 0.001.

The advantage for SCIT in treating coughing is real, but the effect size (eta-squared) is only 0.025, meaning that it only accounts for 2.5% of the variance in pre- versus posttreatment differences, which is not much. Therefore, it can be concluded that SCIT and SLIT exhibit similar efficacy. The advantage of SLIT in treating wheezing may have been influenced by our own bias of suggesting SLIT use to asthmatic patients as a safer treating modality. It is therefore more likely that patients with higher symptom scores were present in the SLIT group.

Our findings demonstrate that SLIT is not only effective in controlling symptoms in nasal allergy patients with or without asthma, in decreasing medication use in such patients, and in improving parameters of pulmonary function, but it also appears that SLIT is as effective as SCIT

These findings are in agreement with those published in the European literature [26, 27] but certainly this presentation lacks the scientific validity of other reports [9] that present a prospective, randomized, controlled study; therefore this presentation we hope will serve as a stimulus for centers with the capability to undertake such a study to continue with this line of research. This would help the FDA to finally recognize SLIT as an effective and safe treatment modality. If SLIT became an FDA-approved treatment modality (and hopefully) reimbursed by insurance companies many more patients might be receptive to immunotherapy which is a treatment capable of altering the immunological mechanisms responsible for the development of allergic conditions [28].

PF values for asthma control should be taken as a guideline only because the predicted lung function has a high degree of variability with significant differences in PF values according to presence or not of lung disease, smoking, age, sex, and even patient's social environment [29–31].

Having the advantage of providing results quickly, and requiring little training (from the patient as well as from the technical staff), the PFM device is useful to monitor progress during immunotherapy [32]. It is most useful when the changes in PF values are compared to the initial value of each patient, recorded at the time of treatment initiation [32]. For the purpose of this study individual improvement with therapy is not reported, but rather an overall trend, thus the use of PFM provides a gross indicator of change.

Immunotherapy is administered over a long period of time. Some of our patients were children, and it is expected they grow during treatment. Certainly using a PFM as a tool to determine improvement in pulmonary function adds uncertainty as to whether the improvement in PF value is related to clinical improvement or to the growth of the patient during treatment. In this study the number of young patients was not large. On the other hand we have demonstrated that the PF value in patients treated by immunotherapy increases regardless of age or asthmatic condition [32].

In our experience, the use of SLIT with multiple antigens has enabled us to treat patients that otherwise would have not received immunotherapy, or would have not continued to receive immunotherapy, like asthmatic patients with poorly controlled asthma, patients that had severe arm reactions, very young patients to whom it is difficult to administer shots or patients whose schedules prevent them from being compliant.

## 5. Conclusions

These results suggest that SCIT and SLIT exhibit similar efficacy. SLIT objectively improves symptom scores for asthma and AR while decreasing medication usage of allergy medications and SABAs.

Given the increased risk and difficulty in treating asthmatic and young patients, these results would suggest that SLIT should be considered as the main treatment modality for these patients, considering SCIT only for treatment failures.

The results of this study are in agreement with the European literature and therefore would support the inclusion of SLIT in the routine management of the allergic disease.

## Figures and Tables

**Table 1:** Allergy test panels.

Dust, dander. and epidermals	Molds	Trees	Grasses	Weeds
Mite pteronyssinus	Alternaria	Ash	Bermuda	Cocklebur
Mite farinae	Aspergillus	Beech	Johnson	English Plantain
Dog	Cladosporium	Birch	Timothy	Goldenrod
Cat	Curvularia	Box Elder		Lambs Quarters
Roach americana	Epicoccum	Elm		Pigweed
Roach germanica	Fusarium	Hickory		Ragweed
	Helminthosporium	Oak		Sagebrush
	Mucor	Sycamore		Sheep Sorrel
	Penicillium			
	Pullularia			

**Table 2:** Symptom Results.

Symptom	No. of patients	Before (mean)	After (mean)	*P* value of *t*-test	Significance of SCIT/SLIT × before/after interaction
Runny nose SCIT	47	2.1	0.7	<0.001	Not significant
Runny nose SLIT	34	1.8	0.5	<0.001	
Sneezing SCIT	47	2.0	0.8	<0.001	Not significant
Sneezing SLIT	39	1.9	0.8	<0.001	
Nasal obstruction SCIT	48	2.4	0.8	<0.001	Not significant
Nasal obstruction SLIT	40	2.2	0.9	<0.001	
Itchy ears SCIT	38	1.5	0.5	<0.001	Not significant
Itchy ears SLIT	30	1.3	0.5	<0.001	
Itchy eyes SCIT	46	1.9	0.7	<0.001	Not significant
Itchy eyes SLIT	37	1.8	0.7	<0.001	
Cough SCIT	46	1.7	0.4	<0.001	Greater improvement for SCIT (*P* = 0.037)
Cough SLIT	30	1.2	0.4	<0.001	
SOB SCIT	6	1.4	0.5	0.041	Not significant
SOB SLIT	9	2.0	0.8	0.005	
Wheezing SCIT	4	1.3	0.5	0.042	Greater improvement for SLIT. (*P* = 0.024)
Wheezing SLIT	7	2.5	0.3	0.001	

**Table 3:** Medication use.

Medication	no. of patients	Before (mean)	After (mean)	*P*-value of *t*-test	Significance of SCIT/SLIT × before/after interaction
Pills in SCIT	37	2.0	0.5	<0.001	Not significant
Pills in SLIT	25	1.5	0.4	<0.001	
INS in SCIT	28	1.5	0.3	<0.001	Not significant
INS in SLIT	26	1.2	0.2	<0.001	
SABA in SCIT	6	1.6	0.9	0.047	Not significant
SABA in SLIT	9	1.1	0.2	0.010	

**Table 4:** Peak Flow Meter determinations. (L/m = litters per minute).

PFM (L/m)	no. of patients	Before (mean)	After (mean)	*P*-value of *t*-test	Significance of SCIT/SLIT × before/after interaction
PFM in SCIT	44	368	467	<0.001	Not significant
PFM in SLIT	36	323	422	<0.001	
